# Involvement of 4-pentenoic acid in causing quality deterioration of nettle silage: study of antibacterial mechanism

**DOI:** 10.1128/spectrum.02667-24

**Published:** 2025-04-30

**Authors:** Rongzheng Huang, Yuxin Chai, Shuangming Li, Yongcheng Chen, Shu'an Jia, Chunhui Ma, Fanfan Zhang

**Affiliations:** 1 Grassland Science, School of Animal Technology, Shihezi University 70586, Shihezi, Xinjiang, China; Fujian Agriculture and Forestry University, Fuzhou, Fujian, China

**Keywords:** *Pediococcus pentosaceus*, nettle silage, 4-pentenoic acid, ammonia

## Abstract

Nettle showed several benefits for animals as an unconventional feed source, but it is difficult to ensile in order to be better utilized. Recently, nettle has been utilized to alternate alfalfa as ruminant feed, but it is usually poor in ensiling quality. To determine the endogenous component influencing ensiling, we investigated the effect of *Pediococcus* on the characteristics of nettle silage and the mechanism of action of these substances against *Pediococcus*. Inoculation with *Pediococcus pentosaceus* decreased the relative abundance of *Clostridium sensu stricto 15* by 85.95% in the middle fermentation stage of nettle silage (30 d), with a 24.74% decrease in the ammonia content (*P*＜0.05). In the correlation analysis, 4-pentenoic acid showed an extremely significant negative correlation with *Pediococcus* spp. in nettle silage (*P* < 0.001). After 4-pentenoic acid treatment, the most downregulated proteins were involved in the ribosome pathway (30 differentially expressed proteins), and then in the glycolysis/gluconeogenesis, pyruvate metabolism, and fatty acid synthesis pathways (particularly for *acc*A, *acc*D, and *fab*G). The mechanism of action of 4-pentenoic acid against *P. pentosaceus* mainly involves inhibition of fatty acid synthesis and decreased the expression of acid tolerance proteins. The present study will give new insights into silage fermentation and provide new clues for better ensiling of nettle.

**IMPORTANCE:**

Nettle has attracted the attention of scientists due to its several benefits for animals as non-conventional feed sources. However, as for challenge, nettle is difficult to ensile (poor quality), which is an obstacle for nettle use. In the present manuscript, we investigated the effect of *Pediococcus* on the characteristics of nettle silage and clarified the mechanisms of 4-pentenoic acid against *Pediococcus*. Our findings suggested that *P. pentosaceus* could improve nettle silage quality at a significant level through decreased production of ammonia (decline percentage was 21.41%–31.73%) during ensiling, while it could not well improve the quality of nettle silage due to the interference effect of 4-pentenoic acid as an antibacterial substance. The mechanism of 4-pentenoic acid against *P. pentosaceus* was mainly through inhibition of fatty acid synthesis (fabG) and expression of acid tolerance protein (accA), resulting in destruction of the cell wall in *P. pentosaceus.* Our finding could give a new clue for better use of nettle silage.

## INTRODUCTION

The scarcity of animal feed resources has driven the recent development of unconventional feed sources (1). Among these, nettle has gained attention because of its multifaceted benefits to animals. These advantages include improved animal performance and production, enhanced rumen health, accelerated growth, and increased disease resistance (2).

Ensiling is crucial for preserving forage nutrition (3). However, ensiling legumes such as alfalfa presents challenges because of their high buffer capacity and protein content, as well as their low levels of water-soluble carbohydrates (WSCs). Nevertheless, the addition of lactic acid bacteria (LAB) and molasses (a source of WSCs) can mitigate nutrient loss in alfalfa silage (4). Compared with ensiling of alfalfa, that of nettle (a member of the *Urticaceae* family) is even more challenging. Despite inoculation with *Lactiplantibacillus plantarum* (a strain of homofermentative LAB) at an adequate fermentation substrate level (>5% dry matter [DM] WSC content), the ensiling process was not facilitated (5), possibly because of the strong inhibitory effects of nettle on bacteria such as *Enterococcus* spp., *Enterobacter* spp., and *Lactiplantibacillus* spp. (6). Silage fermentation is typically initiated by spherical LAB species, such as *Enterococcus* spp. and *Pediococcus* spp. Over time, rod-shaped LAB species, such as *Lactiplantibacillus* spp., become dominant in well-preserved silage (7). The predominant factor contributing to this challenge in nettle ensiling is likely the limited activity of *Lactiplantibacillus* spp. observed in our previous study (8).

Recently, researchers showed that inoculation with *Pediococcus sp*. can improve the quality of legume silage by increasing lactic acid (LA) production and reducing pH levels (9). The relative abundance of spherical LAB, such as *Pediococcus* spp., remains stable in nettle silage (8). However, various substances, including condensed tannins and flavonoids, can inhibit *Pediococcus sp*. activity during ensiling or under specific conditions (10, 11). We investigated whether *Pediococcus sp*. can influence fermentation characteristics or overcome these challenges in nettle silage. In addition, we aimed to identify the factors influencing these bacteria.

## MATERIALS AND METHODS

### Strain isolation

*Pediococcus sp*. strains were isolated from nettle silage under natural fermentation conditions. Bacterial isolation was performed as described by Adhikari et al. (2001) (12). Bacterial fluids were collected for 16S rRNA sequencing, which was performed by Sangon Biotechnology Co., Ltd. (Shanghai, China). The primers used for polymerase chain reaction amplification were as follows: 27F: 5ʹ-GCAGAGTTCTCGGAGTCACGAAGAGTTTGATCCTGGCTCAG-3ʹ and 1492R: 5ʹ-AGCGGATCACTTCACACAGGACTACGGGTACCTTGTTACGA-3ʹ. The nucleotide sequence data of the bacteria were deposited in the National Center for Biotechnology Information under accession number “OL455604” (accessed on November 19, 2021). The phylogenetic evolution tree of the bacteria is shown in Fig. S1, and the specific methods used for phylogenetic analysis can be found in the Supporting Information. All reagents used in this study were procured from Sigma-Aldrich (St. Louis, MO, USA), unless otherwise specified.

### Silage preparation

Nettle (*Urtica cannabina*) was harvested on 20 September 2022, from the wild in Shawan County in the central mountains of Tianshan, Xinjiang, China (E 84°58′–86°24′; N 43°26′−45°20′). The nettle was allowed to wilt at approximately 370 g/kg fresh weight. The samples were chopped into stalks of 2 cm using a forage cutter. *Pediococcus* spp. (accession number: OL455604) was isolated from the natural fermentation of nettle silage and enriched with MRS liquid medium. After determining the LAB count using the plate counting method, *P. pentosaceus* was evenly sprayed on each bag of the silage surface and completely mixed for storage. Following manual mixing, approximately 1.0 kg samples were packed into polyethylene plastic bags equipped with a one-way air extraction valve (23 × 30 cm). The experimental groups were inoculated with *P. pentosaceus* at a concentration of 1 × 10^6^ colony-forming units per gram fresh weight, whereas an equal volume of sterilized water was sprayed as a control in another set of bags. These bags were sealed using a vacuum sealer and stored at 24°C. There were four fermentation period treatments (7, 15, 30, and 60 hours), two inoculation treatments, and five replicates for each treatment, resulting in 40 bags of samples.

### Characteristics analysis of silage

After different fermentation periods, 200 g samples were collected and dried at 65°C for 48 hours to determine the DM content. All samples were crushed through a 1 mm sieve to determine other parameters. The total nitrogen content was determined using an automatic Kjeldahl nitrogen analyzer (K9840, Hanon Co., Ltd., Shandong, China), and the cured protein content was calculated as described by the Association of Official Agricultural Chemists. The WSC content was determined as previously described (13). Fresh silage samples (20 g) were used to analyze fermentation characteristics. The LA, acetic acid (AA), propionic acid (PA), pH, and ammonia (AN) levels were measured as described previously (8).

### Sequencing analysis of bacterial communities in nettle silage

Samples were selected after 7, 30, and 60 days of ensiling. Total DNA was extracted from each silage sample using a commercial DNA Kit (FastDNA Spin Kit for Soil, MP Biomedicals, Irvine, CA, USA). PCR amplification was performed by targeting the V3–V4 regions of the 16S rDNA gene using specific primers (338F: 5ʹ-ACTCCTACGGGAGGCAGCAG-3ʹ; 806R: 5ʹ-GGACTACHVGGGTWTCTAAT-3ʹ). The amplicons were purified and analyzed as previously described (14). Three replicates were included for each sample, and a pooled mixture of these replicates was subjected to sequencing. The obtained sequences were deposited in the National Center for Biotechnology Information database.

### Metabolite analysis in naturally fermented nettle silage

The following method was followed based on our previous study (8). Briefly, 50 g of the fresh sample was collected on each natural fermentation day (after 7, 30, and 60 days of ensiling) of nettle silage, with five replicates for each silage. Specific methods can be found in the **Supporting Information**.

### Minimum inhibitory concentration of antibacterial substances on *P. pentosaceus*

Potential antibacterial substances against *P. pentosaceus* were selected based on the results of the correlation analysis between bacteria and metabolites in nettle silage under natural fermentation conditions (Fig. S2). The minimum inhibitory concentrations of the substances against *P. pentosaceus* were determined using the broth dilution method (15). Briefly, after filter membrane sterilization (aperture, 0.45 µm), the substance was diluted to 1–40 mg/mL. The bacterial solution (1 mL in MRS broth, grown for 3.5–4 hours) was diluted to 10^6^ colony-forming units per milliliter. The bacterial solution (50 µL) mixed with 50 µL of the substance solution was added to 96-well microporous plates, followed by incubation at 37°C for 24 hours.

### Growth of *P. pentosaceus* under antibacterial substance stress

Bacteria were cultured on MRS broth with and without addition of 0%, 0.2%, 0.4%, 0.6%, 0.8%, and 1.0% (vol: vol) antibacterial substances for 72 hours at 37°C. The absorbance was determined at 600 nm and pH after 12, 24, 36, 48, and 72 hours of incubation.

### Transmission electron microscopy analysis

Based on the growth results, the selected substances exhibited the greatest inhibitory effects on *P. pentosaceus*. Bacteria were cultured on MRS broth with 0.8% (vol: vol) 4-pentenoic acid for 8 hours (logarithmic phase) at 37°C. The bacterial cells were collected after centrifugation (8,000 rpm for 5 minutes) and washed twice in 0.1 M phosphate-buffered saline (pH 7.0). Bacterial fixation and dehydration were performed as described above (16). The samples were embedded in an ultramicrotome (EM-UC7, Leica Co., Ltd., Wetzlar, Germany) to obtain 70–90 nm thin sections, which were double-stained with uranyl acetate and lead citrate and analyzed using a transmission electron microscope (HT 7800, Hitachi Co., Ltd., Japan).

### Proteomic analysis of *P. pentosaceus* under antibacterial substance stress

Bacteria were cultured on MRS broth with and without 0.8% (vol: vol) 4-pentenoic acid for 8 hours at 37°C. The bacterial cells were collected after centrifugation (8,000 rpm for 5 minutes) and washed twice in 0.1 M phosphate-buffered saline (pH 7.0). Parallel reaction monitoring analysis was performed to validate the proteomic results. The specific methods used for proteomic analysis are described in the **Supporting Information**.

### Statistical analysis

The characteristic data of the two silage groups were analyzed using Student’s *t*-test. Data were analyzed using SPSS 22 Statistics (SPSS, Inc., Chicago, IL, USA). Differences between treatments were considered significant at *P* < 0.05. Bioinformatic analysis of the silage microbiota and metabolites was performed using the Majorbio Cloud platform (https://cloud.majorbio.com). Specific methods can be found in the **Supporting Information**.

## RESULTS

### Characteristics of nettle silage after inoculation with *P. pentosaceus*

The characteristics of nettle after wilting were 37.56% of dry matter, 16.57% DM of the cured protein, 7.49% DM of WSC, and 7.23 of pH. As shown in Table 1, during fermentation, the DM content was lower in the PP (inoculation with *P. pentosaceus* in nettle silage) samples than in the control after 7 days of ensiling (*P* < 0.05). The pH was lower in PP plants than in control plants after 7 and 60 days of ensiling (*P* < 0.05). The LA content was highest, but the AA content was lowest in PP-treated plants compared with that in control plants after 7 days of ensiling (*P* < 0.05). The AN content was lower in the PP group than in the control group after 30 and 60 days of ensiling (*P* < 0.05). The 4-pentenoic acid content was higher after 7 days of fermentation than after the other fermentation days (Fig. S3, *P* < 0.05).

**TABLE 1:** The fermentation characteristics of nettle silage % DM^*a*^

Items	Ensiling days	Treatment		SEM	*P* value
		CK	PP		
DM (% fresh weight)	7	35.63b	37.72a	0.012	＜0.001
	15	35.68	35.27	0.010	0.360
	30	35.44	33.89	0.012	0.160
	60	36.32	35.08	0.011	0.083
CP	7	16.23	16.08	0.121	0.078
	15	15.82	15.99	0.164	0.053
	30	15.26	15.19	0.450	0.069
	60	13.27	13.23	0.384	0.064
WSC	7	2.91	2.80	0.261	0.618
	15	1.61	1.60	0.143	0.997
	30	0.68	0.62	0.046	0.339
	60	0.69	0.70	0.064	0.724
pH	7	7.92a	7.64b	0.132	＜0.001
	15	7.77	7.87	0.083	0.263
	30	8.35	8.37	0.058	0.059
	60	8.46a	8.01b	0.132	＜0.001
LA	7	2.60	2.80	0.227	0.18
	15	3.27	3.58	0.459	0.273
	30	ND	ND	-	-
	60	ND	ND	-	-
AA	7	1.85a	1.45b	0.218	0.027
	15	1.92	2.05	0.360	0.238
	30	ND	ND	-	-
	60	ND	ND	-	-
PA	7	0.06	0.05	0.015	0.414
	15	0.09	0.09	0.032	0.097
	30	ND	ND	-	-
	60	ND	ND	-	-
AN (%TN)	7	1.06	1.48	0.25	0.167
	15	3.59	4.12	0.33	0.254
	30	6.71a	5.05b	0.55	0.039
	60	9.00a	6.14b	0.11	＜0.001

^*a*^ CK: control; PP: inoculation with *P. pentosaceus*; DM: dry matter; CP: cured protein; WSC: water-soluble carbohydrates; LA: lactic acid; AA: acetic acid; PA: propionic acid; AN: ammonia nitrogen; TN: total nitrogen;ND means “not detected”; “-” means the data have no statistical analytical significance as “not detected”.

### Bacteria community analysis in nettle silage after inoculation with *P. pentosaceus*

**Fig 1:**
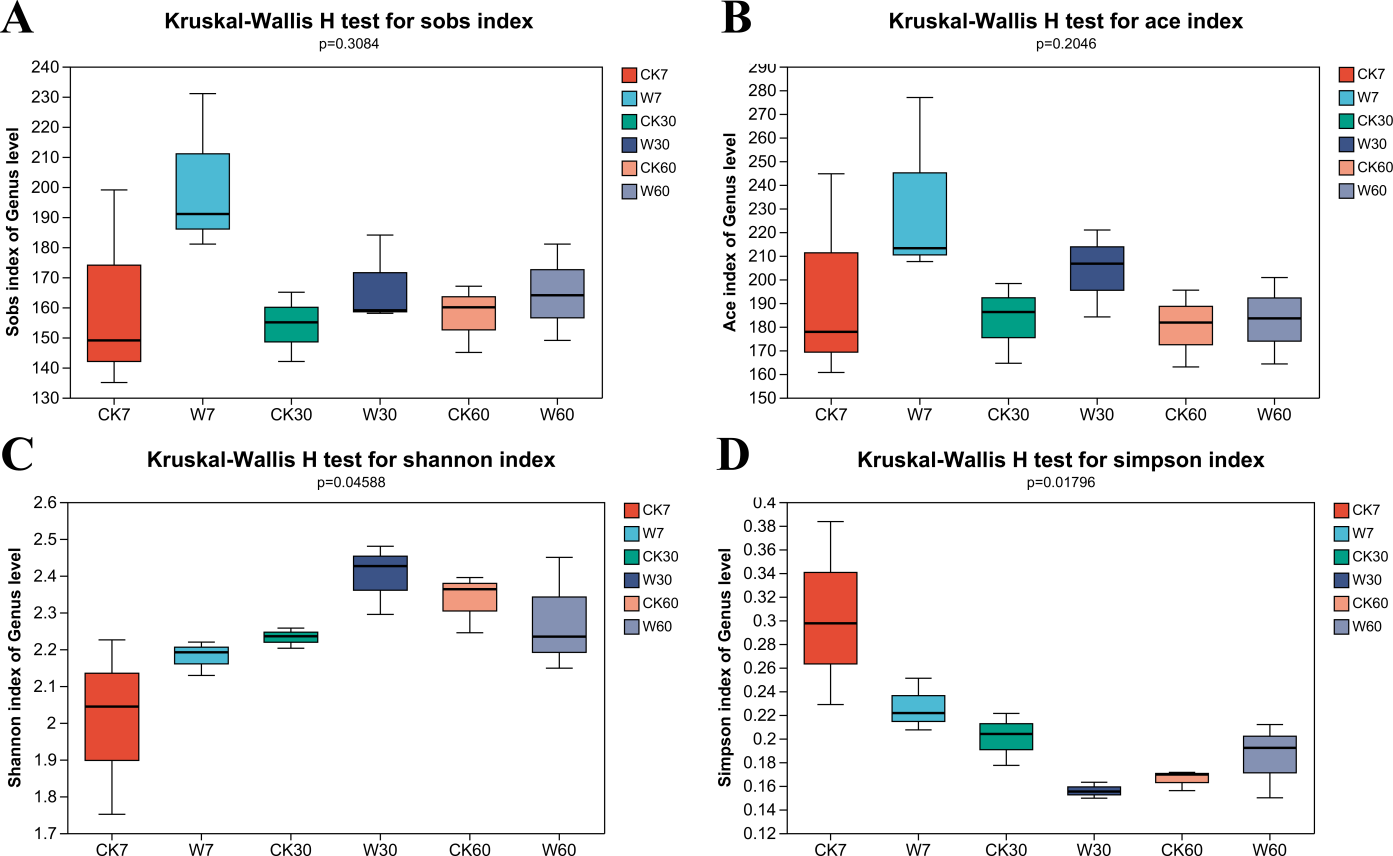
Alpha diversity of the bacterial community for nettle silage. A. Sobs index; B. ACE index; C: Shannon index; D: Simpson index. CK 7: control group after 7 days of ensiling; CK 30: control group after 30 days of ensiling; CK 60: control group after 60 days of ensiling; PP 7: inoculation with *P. pentosaceus* after 7 days of ensiling; PP30: inoculation with *P. pentosaceus* after 30 days of ensiling; PP60: inoculation with *P. pentosaceus* after 60 days of ensiling.

Based on the results of the alpha diversity analysis of the bacteria, as shown in Fig. 1, only the Shannon and Simpson indices significantly differed between the control and treated groups, with higher values in the treated groups for the Shannon index and lower values in the control groups for the Simpson index after 7 and 30 days of ensiling, respectively (*P* < 0.05).

**Fig 2:**
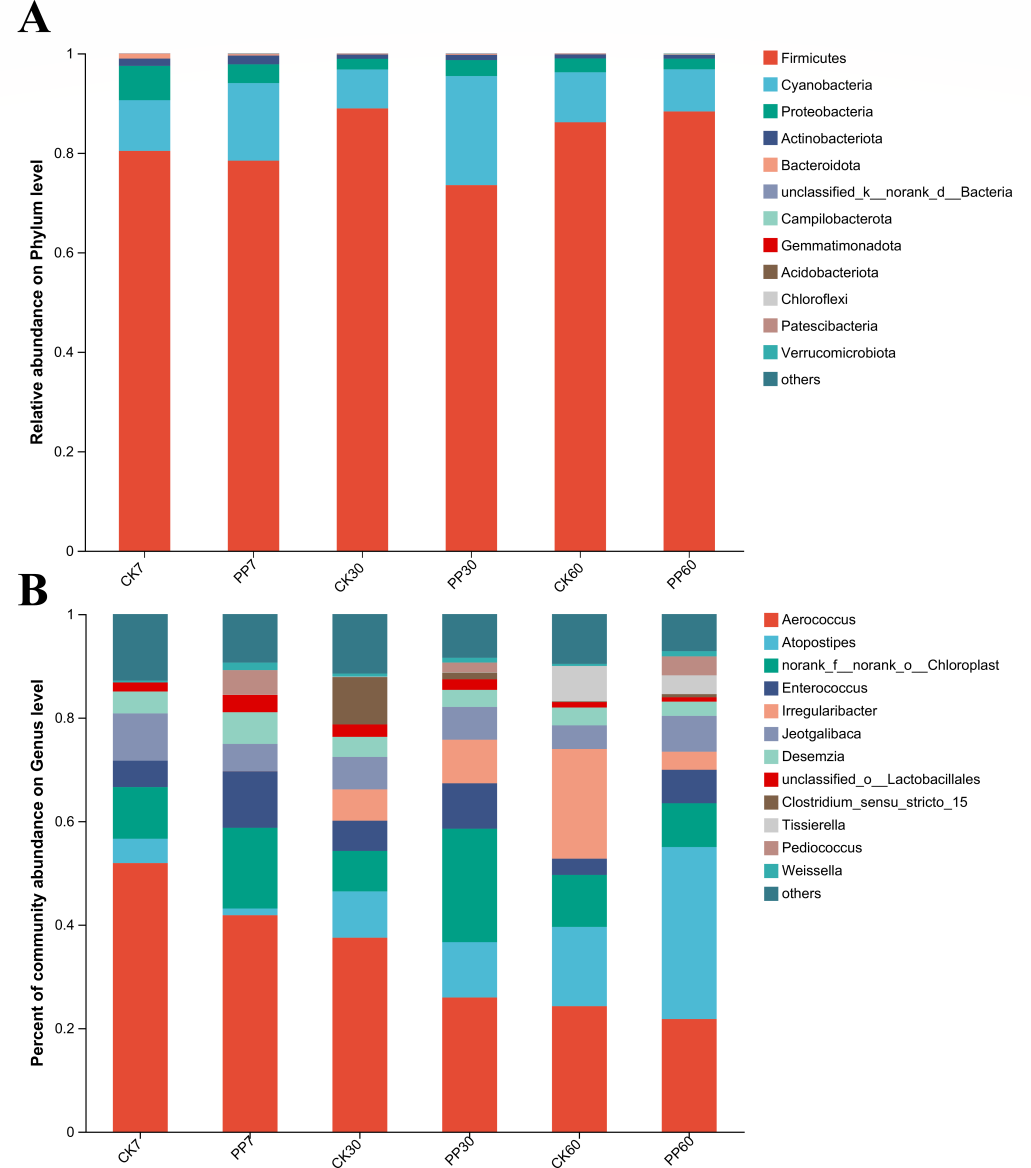
Effects of inoculation with *Pediococcus pentosaceus* on bacterial communities at the phylum and (**B**) genus levels. CK 7: after 7 days of ensiling in the control group; PP 7: after 7 days of ensiling in the *P. pentosaceus* inoculation group.

As shown in Fig. 2A, at the phylum level, Firmicutes were dominant in both the control and PP-treated groups throughout the ensiling process. As shown in Fig. 2B, at the genus level, *Aerococcus* was dominant, followed by *Atopostipes* and *Enterococcus* during ensiling. The relative abundance of *Aerococcus* was lower in the PP group than in the control group (21.75%–41.82% vs 24.24%–51.90%, *P* < 0.05) throughout the ensiling process. The relative abundance of *Atopostipes* was lowest in PP plants compared with in control plants after 7 days of ensiling (1.30% vs 4.70%, *P* < 0.05), but increased in PP plants with prolonged ensiling time (*P* < 0.05). The relative abundance of *Enterococcus* was higher in the PP-treated group than in the control group (6.46%–10.93% vs 3.13%–5.83%, *P*＜0.05) during the entire ensiling process. The relative abundance of *Irregularibacter* was higher in the PP group than in the control group after 7 and 30 days of ensiling (*P* < 0.05) but was lowest in the PP-treated group compared with in the control group, when the ensiling time was prolonged to 60 days (*P* < 0.05). The relative abundance of *Pediococcus* was higher in the PP-treated group than in the control group (1.86%–4.76% vs 0.00%–0.0027%, *P*＜0.05) throughout the ensiling process.

**Fig 3:**
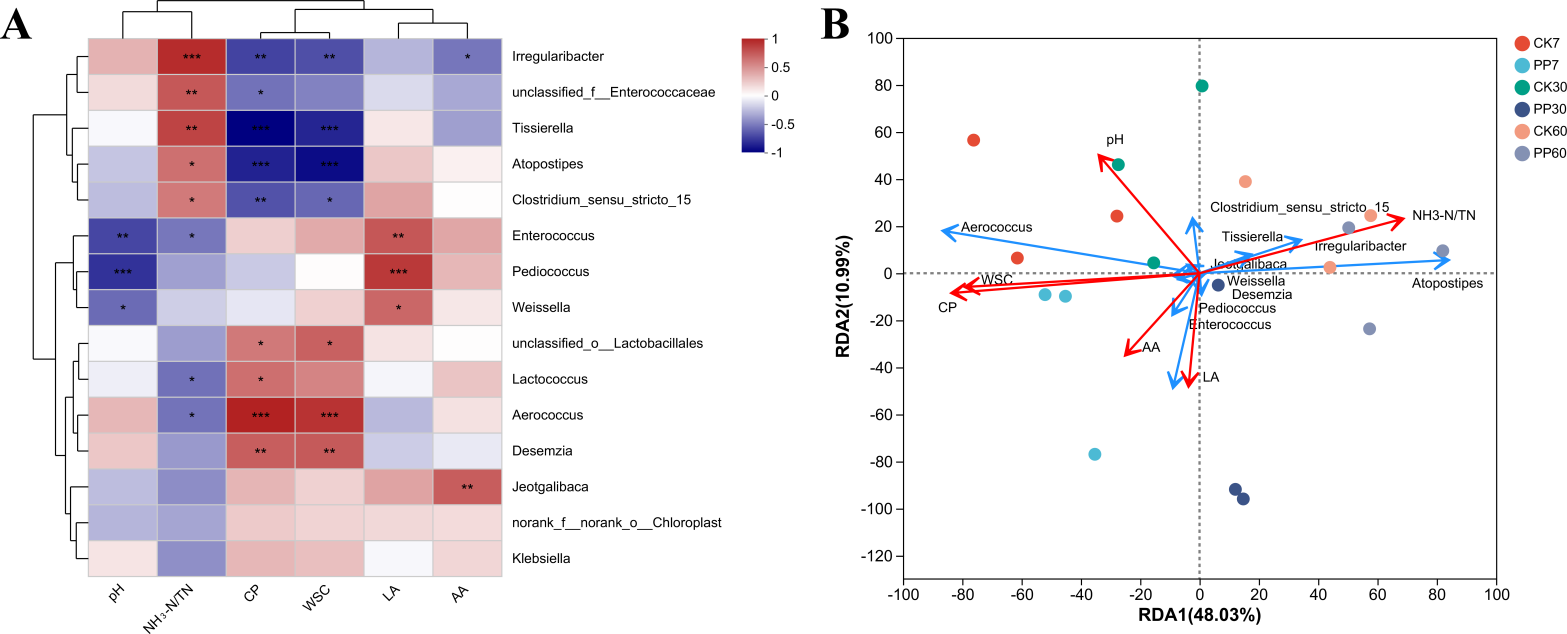
Effect of inoculation with *Pediococcus pentosaceus* on (**A**) the correlation between bacteria and fermentation characteristics and (**B**) redundancy analysis (RDA) in nettle silage. CK 7: after 7 days of ensiling in the control group; PP 7: after 7 days of ensiling in the *P. pentosaceus* inoculation group. *0.01＜*P*＜0.05; **0.001＜*P*＜0.01; ***P＜0.001.

As shown in Fig. 3A and B, there was a significantly positive correlation between *Enterococcus*, *Pediococcus*, and *Weissella* bacteria and LA (*R*^2^ = 0.6243, 0.7431, and 0.5624, respectively, *P*＜0.05), whereas these bacteria both showed a significantly negative correlation with pH (*R*^2^ = −0.6532,–0.7218, and −0.5273, respectively, *P*＜0.05). *Irregularibacter* and *Clostridium_sensu_stricto_15* were significantly positively correlated with ammonia (*R*^2^ = 0.7920 and 0.4917, respectively; *P* < 0.05), whereas both *Enterococcus* and *Lactococcus* showed a significantly negative correlation with ammonia (*R*^2^ = −0.4881 and −0.5080, respectively; *P* < 0.05).

### Mechanism of 4-pentenoic acid against *P. pentosaceus* growth

**Fig 4:**
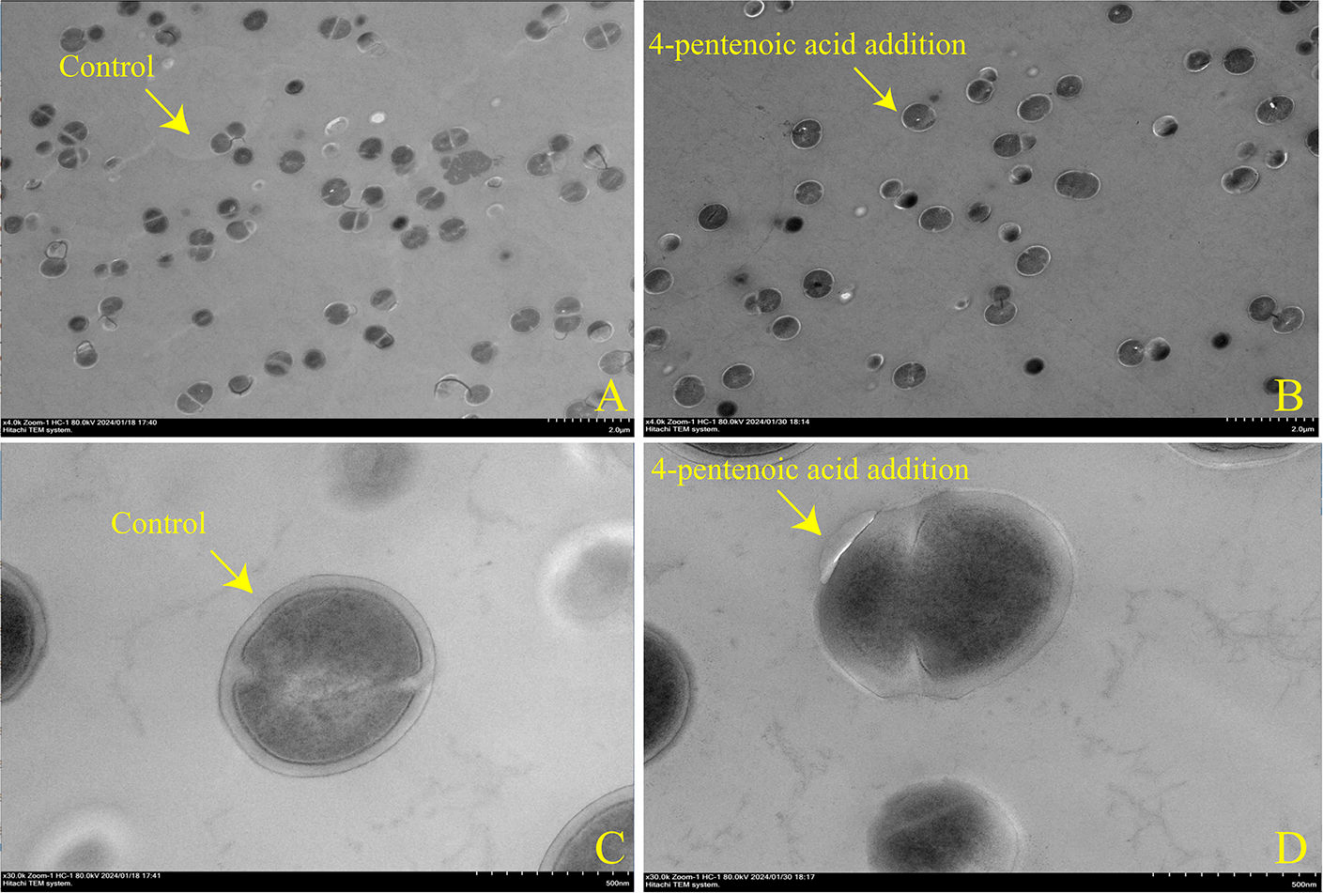
Transmission electron microscopy images showing the effects of 4-pentenoic acid on the morphology of *Pediococcus pentosaceus* at a magnification of × 4.0 K in (**A**) and (**B**) and × 30.0 K in (**C**) and (**D**).

*Pediococcus pentosaceus* was isolated from naturally fermented nettle silage (Fig. S1). As shown in Fig. S2, the correlation between *Pediococcus* and organic acids such as 4-pentenoic acid (*R*^2^ = −0.6323, *P*＜0.001) and gluconic acid (*R*^2^ = −0.2512, *P*＜0.001) was extremely negatively significant during nettle ensiling. The minimum inhibitory concentrations of 4-pentenoic acid and gluconic acid against *P. pentosaceus* were 16 and 32 mg/mL, respectively. The absorbance decreased significantly, but the pH increased as the concentration of 4-pentenoic acid increased in *P. pentosaceus* (*P* < 0.05, Fig. S4). Based on the transmission electron microscopy results shown in Fig. 4, the cell membrane of *P. pentosaceus* was found to be severely damaged under 4-pentenoic acid treatment. Thus, we selected 4-pentenoic acid as an inhibitor of *P. pentosaceus* and further evaluated its antibacterial mechanism.

Results of parallel reaction monitoring showed that 87.50% of the selected differentially expressed proteins (DEPs), such as *accA*, showed the same fold change as observed in the proteomic results (Table S2). There was a strong correlation between the control and treatment groups based on the Pearson’s correlation coefficient (Fig. S5), indicating high repeatability.

**Fig 5:**
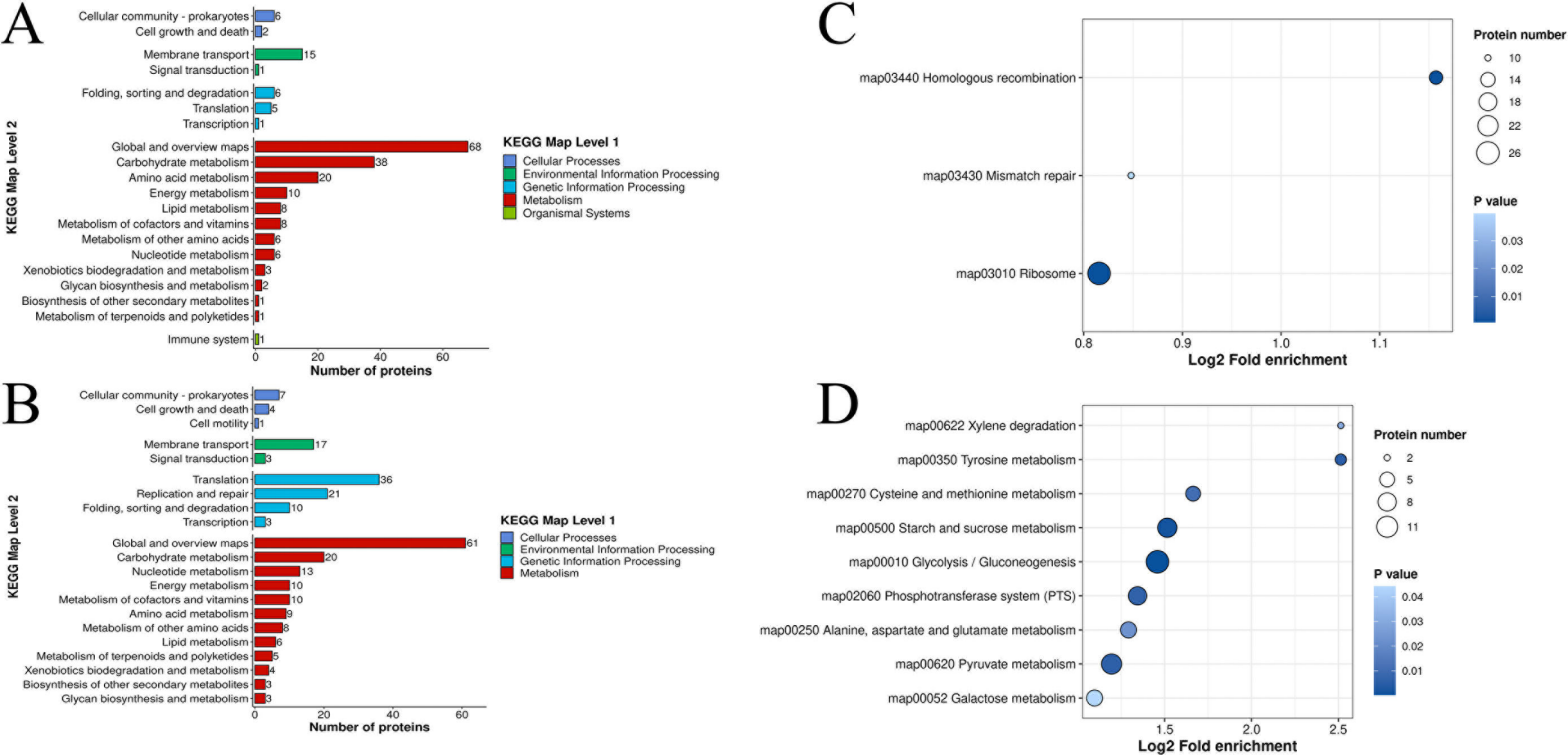
Differentially expressed proteins (DEPs) in *Pediococcus pentosaceus* cells after treatment with 0.8% (**vol: **vol) 4-pentenoic acid for 8 hours were annotated using the Kyoto Encyclopedia of Genes and Genomes database. (**A**) Metabolites: upregulated proteins compared to the control; (**B**) metabolites: downregulated proteins compared to the control. (**C**) Metabolite pathway: upregulated proteins compared to control; (**D**) metabolite pathway: downregulated proteins compared to control.

Based on the Kyoto Encyclopedia of Genes and Genomes (KEGG) annotation, as shown in Fig. 5A and B, 462 DEPs were identified, of which 208 DEPs were upregulated and 254 DEPs were downregulated. Most of these DEPs were annotated to the global and overview maps (32.69% and 24.01% for up- and downregulated DEPs, respectively), followed by carbohydrate metabolism (18.27% and 7.87% for up- and downregulated DEPs, respectively) and translation (14.17% for downregulated DEPs). Most of the upregulated DEPs (Fig. 5C and D) were involved in the glycolysis/gluconeogenesis pathway (6.25%), followed by the pyruvate metabolism pathway (4.81%), starch and sucrose metabolism pathway (4.33%), phosphotransferase system (PTS) pathway (3.85%), ribosome pathway (10.23%), homologous recombination pathway (5.12%), and mismatch repair pathway (3.94%).

**Fig 6:**
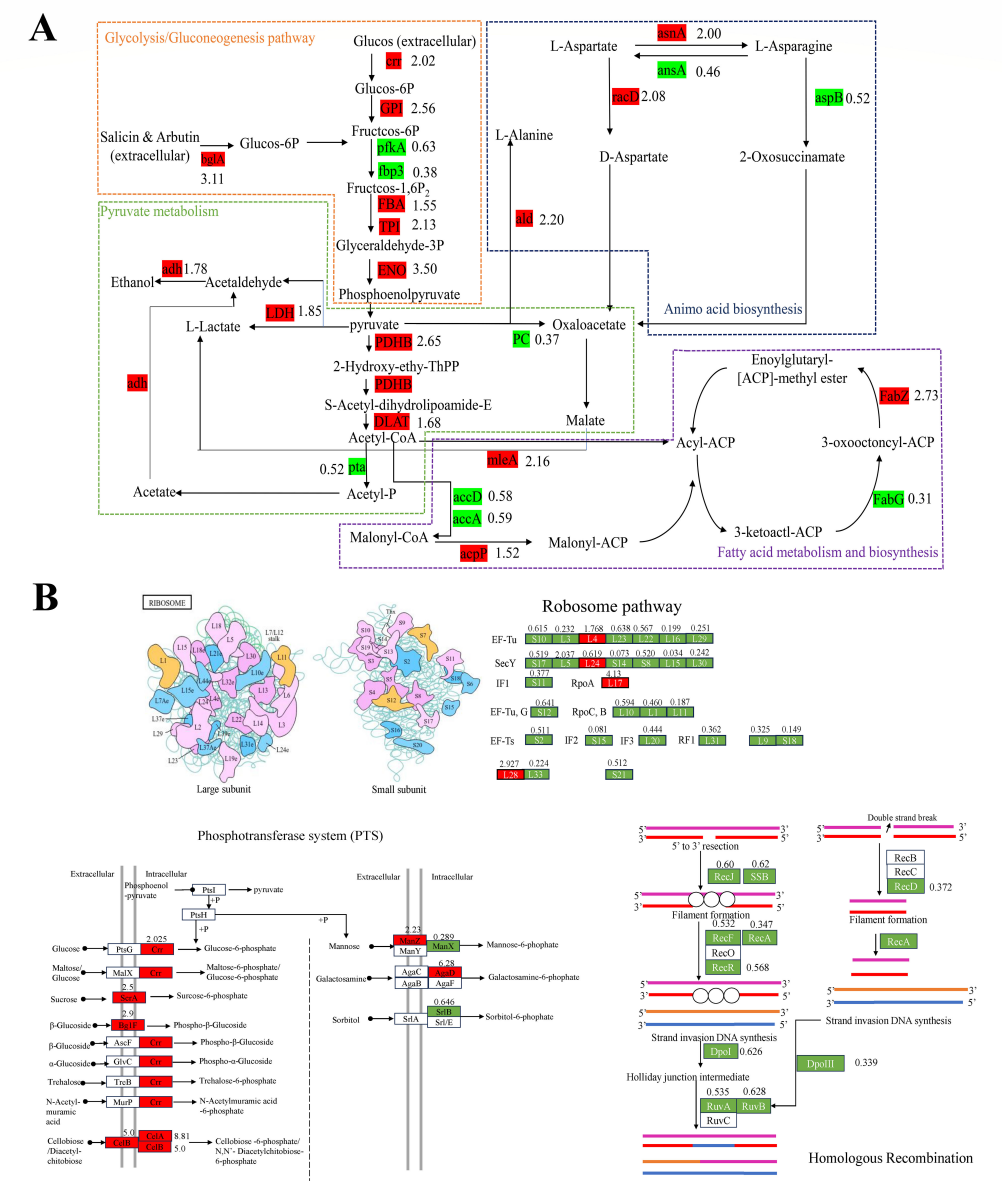
Protein expression profiles of differentially expressed proteins (DEPs) involved in the (**A**) glycolysis/gluconeogenesis pathway, amino acid biosynthesis, pyruvate metabolism, and fatty acid metabolism biosynthesis and (**B**) phosphotransferase system (PTS), ribosome, and homologous recombination pathways in *Pediococcus pentosaceus* after exposure to 0.8% (vol: vol, ≈0.5-fold of the minimum inhibitory concentration [MIC]) of 4-pentenoic acid for 12 hours. The red and green boxes represent up- and downregulated protein expressions, respectively; the number behind the box represents the fold change in the protein expression between control and 4-pentenoic acid-treated cells.

DEPs with significant annotations in the KEGG pathway were as follows (Fig. 6):

#### Glycolysis/gluconeogenesis

Six DEPs were upregulated (crr, GPI, *bgl*A, FBA, TPI, and ENO) and two (*fbp* and *pfk*A) were downregulated after treatment with 4-pentenoic acid.

#### Pyruvate metabolism

Four DEPs were upregulated (PDHB, DLAT, LDH, and *adh*) and two DEPs were downregulated after treatment with 4-pentenoic acid (PC and *pta*).

#### PTS

Seven DEPs were upregulated (crr, scrA, bglF, *cel*A, *cel*B, *manZ*, and *agaD*) and two DEPs were downregulated (*manX* and *srlB*).

#### Fatty acid metabolism and biosynthesis

Three DEPs were upregulated (*Fab*Z, *mle*A, and *acp*P) and three DEPs were downregulated after treatment with 4-pentenoic acid (*acc*D, *acc*A, and *Fab*G).

#### Ribosome

Four DEPs were upregulated (*rpl*D, *rpm*B, *rpl*X, and *rpl*Q); 26 DEPs were downregulated (*rps*B, *rpl*A, *rpm*E2, *rpl*I, *rps*Q, *rpl*K, *rpl*O, *rpl*W, *rpl*V, *rps*K, *rps*H, *rpm*C, *rps*J, *rps*N, *rpl*C, *rpl*J, *rps*R, *rpl*P, *rps*Z, *rpm*G, *rpl*E, *rps*U, *rpm*D, *rps*O, *rps*L, and *rpl*T); four DEPs were upregulated (*rp1*D, *rpm*B, *rp1*X, and *rp1*Q).

#### Homologous recombination

Thirteen DEPs were downregulated (*pol*C, *rec*R, *rec*A, *ruv*B, *dna*X, ruvA, *hol*A, *rec*F, *hol*B, *rec*D, *rec*J, *pol*A, and *ssb*).

## DISCUSSION

### Characteristics of nettle silage inoculated with *P. pentosaceus*

Inoculation with *P. pentosaceus* can increase the DM content in alfalfa silage (17). A ratio of LA-to-AA <3 indicates heterolactic fermentation during ensiling (18), in which LAB such as *Lactobacillus buchneri* can convert WSC into LA, AA, and CO_2_, resulting in a decrease in the DM. In the present study, inoculation with *P. pentosaceus* did not change the fermentation type or lead to a LA-to-AA ratio <3, but the content of AA decreased by 21.62% after 7 days of ensiling, indicating that *P. pentosaceus* can reduce DM loss during the early stage of ensiling fermentation.

AN is an important indicator of silage quality as it can reflect the level of protein degradation, typically accounting for less than 100 g/kg DM, indicating that silage is well-preserved (19). In this study, inoculation with *P. pentosaceus* decreased AN production during nettle ensiling. AN is correlated with undesirable bacteria (such as *Clostridium* and *Enterobacter*) and plant proteolytic enzymatic activity, both of which are inhibited at a pH below 4.6 (20). However, we showed that the pH was >7 after inoculation, indicating that another factor was responsible for the reduction of AN during ensiling. Some species of *Pediococcus* can significantly decrease AN production (87.48% decline) in *Escherichia coli* (21). Thus, our results suggest that *P. pentosaceus* can inhibit undesirable bacterial activity during nettle ensiling.

### Effect of inoculation with *P. pentosaceus* on the bacterial community of nettle silage

Inoculation with *P. pentosaceus* increases the bacterial diversity during nettle ensiling. *Aerococcus* was dominant in nettle silage, and inoculation with *P. pentosaceus* decreased the relative abundance of *Aerococcus* by 10.27%–30.84% during ensiling. Notably, *Aerococcus* has rarely been detected in silage; only one study detecting *Aerococcus* as a facultative anaerobic bacterium showed a negative correlation with AA but a positive correlation with AN in paper mulberry silage, indicating that AA can inhibit *Aerococcus* activity (22). However, as observed for *Aerococcus*, we showed the opposite trend for paper mulberry silage. Some strains of *Aerococcus* can produce AA ranging from 2.3% to 3.4% in the medium (23). The content of AA in nettle silage was 1.45%–2.05%, indicating that AA did not impact *Aerococcus* activity in nettle silage. Similar to *Aerococcus*, *Atopostipes* was rarely detected in silage. Only one study showed that *Atopostipes* was dominant in silage and produced LA and AA, similar to hetero-lactic acid fermentative bacteria (24). Inoculation with *P. pentosaceus* decreased the relative abundance of *Atopostipes* by 72.44% only in the early stages of nettle ensiling (7 days), which was consistent with the decrease in AA.

Inoculation with *P. pentosaceus* increased the relative abundance of *Enterococcus* by 33.51%–53.52% during nettle ensiling and was stabilized during natural fermentation of nettle silage (8). Our results suggest that inoculation with *P. pentosaceus* led to proliferation of *Enterococcus* during ensiling*. Clostridium_sensu_stricto* is typically regarded as the true *Clostridium* genus and is a prominent member of plant-associated endophytes that produce AA by utilizing several WSCs, such as glucose, lactose, and sucrose (25). Inoculation with *P. pentosaceus* decreased the relative abundance of *Clostridium_sensu_stricto 15* by 85.95% in the middle fermentation stage of nettle silage (30 d), resulting in a 24.74% decrease in the AN content. Thus, the decrease in AA (after only 7 days of ensiling) was mainly attributed to inhibition of *Atopostipes* by *P. pentosaceus* during nettle ensiling. Similar results were observed previously in nettle silage, with *Clostridium_sensu_stricto* showing a positive correlation with AN (26). These results suggest that *P. pentosaceus* can inhibit *Clostridium* fermentation during nettle ensiling.

The relative abundance of *Pediococcus* in silage was greatly increased by *P. pentosaceus* inoculation, indicating that inoculation with *P. pentosaceus* can partly overcome the impact of 4-pentenoic acid, which inhibits the activity of *Pediococcus* during ensiling.

### Mechanism of 4-pentenoic acid against *P. pentosaceus* growth

We first observed that 4-pentenoic acid was present during the natural fermentation of nettle silage and that its content was almost stabilized during ensiling (decreased by 4.48% after 90 days of fermentation), inhibiting *P. pentosaceus* growth *in vitro*. Few studies have focused on inhibiting the activity of 4-pentenoic acid against bacteria; only 4-pentenoic acid inhibited *Pseudomonas fluorescens* growth in the fructose-based medium (5 mM, 30% inhibition of cell growth). The mechanism of this effect requires further study (27).

The expressions of proteins involved in the ribosomal pathway were most affected by AA treatment; these proteins are typically downregulated by acid stress (28, 29). Thus, our results showed that most DEPs were downregulated, but four DEPs were upregulated following treatment with 4-pentenoic acid. These results suggest that 4-pentenoic acid has a stronger effect than AA on *P. pentosaceus*.

#### Role of DEPs

DEPs that were significantly annotated in the KEGG pathway and strongly linked to the 4-pentenoic acid response are discussed below.

#### Glycolysis/gluconeogenesis

Most proteins, such as fructose-1, 6 _p2_ (PfKA), glycerate-1,3p_2_ (TPI), and phosphoenolpyruvate (PgaM, ENO, PGK, and PPH) synthesis-related proteins, were upregulated to optimize the use of carbohydrate energy sources when exposed to acid stress (29). Our results were similar to those of Zhang, except for the results for fructose-1, 6 _p2_ inhibited by 4-pentenoic acid as protein (Pfka) encoded by A0A0Q0TT21 was downregulated. These results suggest that 4-pentenoic acid has a greater impact on the energy metabolism in *P. pentosaceus*.

#### Fatty acid metabolism and biosynthesis

Pyruvate carboxylase encoded by Q03HH9 was the most downregulated protein among the DEPs involved in pyruvate metabolism, followed by phosphate acetyltransferase (*pta*) encoded by A0A1Y0VWZ9. Two proteins involved in malonyl-CoA synthesis (accA and accD encoded by A0A1Y0VWI4 and A0A0Q0Y7Z9, respectively) were also downregulated. Malonyl-CoA is a rate-limiting substrate for fatty acid synthesis and is essential for maintaining cell membrane integrity and energy conservation (30). Furthermore, β-ketoacyl-ACP reductase (FabG) encoded by A0A7T4MYU7 was the most downregulated protein, but FabZ encoded by A0A0R2H9C6 involved in fatty acid synthesis was upregulated. FabG is a key rate-limiting enzyme for fatty acid synthesis, and others, such as FabI protein, are upregulated in response to stress when FabG is inhibited by antibacterial substances (31). In addition, we showed that the cell membranes of *P. pentosaceus* were damaged by 4-pentenoic acid. In contrast to AA stress in *Pediococcus,* in which pyruvate metabolism was inhibited (29), our results showed that 4-pentenoic acid inhibits fatty acid synthesis.

#### PTS

Nine proteins were involved in the PTS pathway, 77.78% of which were upregulated, indicating that the cells had some tolerance to acid stress caused by 4-pentenoic acid. Several studies reported similar results when bacteria or fungi were under acid stress (32, 33). Proteins involved in cellobiose transport (CelA and B, 8.81- and 5.0-fold change, respectively) in PTS showed the greatest upregulation. Cellobiose permease consists of CelA/B/C/D/E, which is the only one of several homologously sequenced permeases of PTS (34). Some *Pediococcus* strains showed the capacity to utilize cellobiose in the medium (35). Our results suggest that *P. pentosaceus* is tolerant to 4-pentenoic acid stress through increased expression of proteins involved in the PTS, which increases the uptake of carbohydrates such as cellobiose.

#### Ribosome

Thirty DEPs were involved in ribosome pathways, 86.67% of which were downregulated by 4-pentenoic acid, indicating that 4-pentenoic acid impacts the ribosome integrity of *P. pentosaceus*. Similar results were observed for some strains of *Pediococcus* under AA stress (29).

Two genes (*acc*A and *acp*P) protected *Pediococcus* from AA stress by maintaining the permeability and fluidity of the cell membrane; *acc*A was more efficient than *acp*P, but both interacted with FabG and FabZ, which are involved in fatty acid synthesis, resulting in an increased fatty acid content in the membrane under acid stress (29). In the present study, *acc*A was downregulated when *P. pentosaceus* was subjected to 4-pentenoic acid stress, indicating that 4-pentenoic acid stress may decrease the tolerance of *P. pentosaceus* to acid stress.

### Conclusion

Inoculation with *P. pentosaceus* decreased ammonia production (from 9.00% to 6.10% of total nitrogen) in nettle silage. However, the quality of nettle silage cannot be improved because of interference by 4-pentenoic acid, which exerts antibacterial effects. The mechanism of action of 4-pentenoic acid against *P. pentosaceus* mainly involves inhibition of fatty acid synthesis (*fabG*) and the expression of acid tolerance protein (*accA*), resulting in destruction of the cell wall in *P. pentosaceus*.

## Data Availability

The data sets supporting the conclusions of this article are available in the National Center for Biotechnology (NCBI) Sequence Read Archive (SRA) under accession numbers “PRJNA1112621” for bacteria (Accessed on 25th May, 2024). http:// www.ncbi.nlm.nih.gov.

## SUPPLEMENTAL MATERIAL

Supplemental material spectrum.02667-24-s0001.docx Supplemental methods; Tables S1 and S2; Fig. S1 to S5.

ASM does not own the copyrights to Supplemental Material that may be linked to, or accessed through, an article. The authors have granted ASM a non-exclusive, world-wide license to publish the Supplemental Material files. Please contact the corresponding author directly for reuse.
