# Supplementary material for: Involvement of 4-pentenoic acid in causing quality deterioration of nettle silage: study of antibacterial mechanism
Source: Microbiol Spectr. 2025 Apr 30;13(6):e02667-24. doi: 10.1128/spectrum.02667-24 (PMC12131780; doi:10.1128/spectrum.02667-24)
Supplement: Supplemental material — Supplemental methods; Tables S1 and S2; Fig. S1 to S5. [file spectrum.02667-24-s0001.docx]

**Supplementary materials**

**Supporting Methods**

**2.1 Strain isolation**

**Phylogenetic analysis**

The amplicons were subjected to nucleotide sequence analysis using BLASTN, followed by phylogenetic tree analysis with MEGA X software. The evolutionary history was inferred using the Neighbour-Joining method, and bootstrap analysis was performed for 1000 replicates (Keerthana and Narayanan, 2021).

**2.5 Metabolite analysis in naturally fermentation of nettle silage**

Metabolites were extracted using 400 µl methanol: water (4:1, v/v) solution. After protein precipitation, the supernatant was collected for Ultra-High Performance Liquid Chromatography-Tandem Mass Spectrometry (UHPLC-MS/MS) analysis. The UHPLC system was equipped with an ACQUITY BEH C18 column (100 mm × 2.1 mm i.d., 1.7 µm; Waters, Milford, MA, USA). The mobile phases consisted of 0.1% formic acid in water (solvent A) and 0.1% formic acid in acetonitrile: isopropanol (1:1, v/v) solution (solvent B). The sample injection volume was 2 µl and the flow rate was 0.4 ml/min. The column temperature was maintained at 40 ℃.

**2.9 Proteomic analysis in *P. pentosaceus* under antibacterial substances stress**

**(1) Total protein extraction**

Take out the samples in the frozen state and put it on ice. Add an appropriate amount of protein lysate (8 M urea, 1% SDS), which contains protease inhibitor to inhibit protease activity. The mixture was treated by ultrasound for 2 min at a low temperature, following splitting for 30 min. After centrifugation at 12000g at 4°C for 30min, the concentration of protein supernatant was determined by Bicinchoninic acid (BCA) method by BCA Protein Assay Kit (Pierce, Thermo, USA). Protein quantification was performed according to the kit protocol.

1. **Protein reductive alkylation and digestion**

Take protein samples 100 μg and add TEAB (Triethylammonium bicarbonate buffer), which the final concentration of TEAB is 100 mM. Then add TCEP (tris (2-carboxyethyl) phosphine) to the final concentration of 10 mM and react for 60 min at 37 °C. Following add IAM (Iodoacetamide) to the final concentration of 40 mM and react for 40 min at room temperature under dark conditions. Add a certain percentage (acetone: sample v/v = 6:1) of pre-cooled acetone to each sample and to settle for 4 h at -20 °C. After centrifugal for 20 min at 10000 g, the sediment was collected and add 100 µL 100mM TEAB solution to dissolve. Finally, the mixture was digested with Trypsin overnight at 37 °C added at 1:50 trypsin-to-protein mass ratio.

1. **Peptide desalination and quantification**

The peptides were vacuum dried, then re-susoended with 0.1% TFA. Samples were desalted with HLB, and vacuum dried. Peptide concentrations were determined by peptide qutification kit (Thermo, Cat.23275). Loading buffer was added to each tube to prepare samples for mass spectrometry analysis, and the concentration of each sample was 0.25 µg/µL.

1. **LC-MS/MS analysis**

Trypsin-digested peptides were analyzed by an EASY nLC-1200 system (Thermo, USA) coupled with a timsTOF Pro2 (Bruker, Germany) mass spectrometer at Majorbio Bio-Pharm Technology Co. Ltd. (Shanghai, China). Briefly, the C18-reversed phase column (75 μm × 25 cm, Ionopticks, USA) as equilibrated with solvent A (A:2 % ACN with 0.1 % formic acid) and solvent B (B: 80 % ACN with 0.1 % formic acid). The peptides were eluted using the following gradient: 0-45 min, 3 %-28 % B; 45-50 min, 28%−44 % B; 50-55 min, 44 %−90 % B; 55−60 min, 90 %-90 % B. The tryptic peptides were separated at a flow rate of 250 nL/min. Peptides were separated by an ultrahigh performance liquid phase system subjected to a capillary ion source and then analyzed by timsTOF Pro2 (Bruker, Germany); the electrospray voltage was 1.5 kV. The peptide parent ions and their secondary fragments were detected and analyzed using high-resolution TOF. The secondary MS scanning range was 100-1700 m/z. Data acquisition on the timsTOF Pro2 was collected using the parallel accumulation serial fragmentation (PASEF) acquisition mode. After the first MS stage, the second MS stage (charge number of the parent ions was 0–5) was recorded using the 10 PASEF mode. A dynamic exclusion time of 24 s was used for the MS/MS scan.

1. **Sequence Database Searching**

MS/MS spectra were searched using MaxQuant version 2.0.3.1 software against database. The highest score for a given peptide mass (best match to that predicted in the database) was used to identify parent proteins. The parameters for protein searching were as follows: tryptic digestion with up to two missed cleavages, carbamidomethylation of cysteines as fixed modification, and oxidation of methionines and protein N-terminal acetylation as variable modifications. False discovery rate (FDR) of peptide identification was set as FDR ≤ 0.01. A minimum of one unique peptide identification was used to support protein identification.

1. **Parallel reaction monitoring (PRM) analysis**

Used the PRM to validated the difference expression protein. Based on results of DEPs through 4D-LabeIfree method (provide by Majorbio Bio-Pharm Technology Co. Ltd., Shanghai, China), we choose sixteen proteins for PRM, as showed in Table S 1. Procedure as followed. First, the proteins in the samples were extracted and enzymatically digested to produce a peptide mixture, and desalted for quantification. Then, equal amounts of enzymatically digested peptides were dissolved in mass spectrometry loading buffer (2 % ACN with 0.1 % formic acid) and analyzed by an EASY nLC-1200 system (Thermo, USA) coupled with a Q Exactive HF-X quadrupole orbitrap mass spectrometer (Thermo, USA) at Majorbio Bio-Pharm Technology Co. Ltd. (Shanghai, China). The peptides were separated by C18-reversed phase column (75 μm×25 cm, Thermo, USA) as equilibrated with solvent A (2 % ACN with 0.1 % formic acid) and solvent B (80 % ACN with 0.1 % formic acid) at the EASY-nLC 1200 liquid phase system. The flow rate was 300 nL/min. The peptides were eluted using the following gradient: 0-64 min, 5 %-23 % B; 64-80 min, 23 %−29 % B; 80-90 min, 29 %-38 % B; 90-92 min,38 %-48 % B; 92-93 min, 48 %-100 % B; 93-120 min, maintain 100 % B. The separated peptides were analyzed by Q-Exactive HF-X (Thermo, USA), detection mode is positive ion; acquisition mode is Parallel Reaction Monitoring (PRM); fragmentation mode is HCD. Full scan ranged from m/z 350 to 1500 with resolution of 60,000 (at m/z 200), an automatic gain control (AGC) target value was 3×10^6^ and a maximum ion injection time was 20 millisecond. PRM scans (PRM MS2 scans) were acquired after each full scan. The secondary resolution is 15000, AGC target value was 1×10^6^ and a maximum ion injection time was 20 millisecond, a normalized collision energy was set as 28 %, isolation window was 2.0 m/z; dynamic exclusion time was 18 s.

**2.10 Statistical analysis**

**(1) Bacteria sequence**

Based on the OTUs information, rarefaction curves and alpha diversity indices including observed OTUs, Chao1 richness, Shannon index and Good’s coverage were calculated with Mothur v1.30.1(Schloss et al., 2009). The PERMANOVA test was used to assess the percentage of variation explained by the treatment along with its statistical significance using Vegan v2.5-3 package. The distance-based redundancy analysis (db-RDA) was performed using Vegan v2.5-3 package to investigate effect of silage characteristics on bacterial community structure. Forward selection was based on Monte Carlo permutation tests (permutations = 9999). Values of the x- and y-axes and the length of the corresponding arrows represented the importance of each silage characteristic parameters in explaining the distribution of taxon across communities.

1. **Metabolite analysis**

The mass spectrometric data were collected using a Thermo UHPLC-Q Exactive Mass Spectrometer equipped with an electrospray ionization (ESI) source operating in either positive or negative ion mode. The optimal conditions were set as follows: Aus gas heater temperature, 400◦C; Sheath gas flow rate 40 psi; Aus gas flow rate 30 psi; ion-spray voltage floating (ISVF), −2,800 V in negative mode and 3,500 V in positive mode, respectively; normalized collision energy, 20–40–60 V rolling for MS/MS. Data acquisition was performed in the Data Dependent Acquisition (DDA) mode. The detection was carried out over a mass range of 70–1,050 m/z. Correlation analysis between bacteria and metabolites in nettle silage (naturally fermentation condition) were determined followed method describe above (Sun et al., 2023).

1. **Proteomic analysis**

Bioinformatic analysis of proteomic data was performed with the Majorbio Cloud platform (https://cloud.majorbio.com). *P*-values and Fold change (FC) for the proteins between the two groups were calculated using R package “t-test”. The thresholds of fold change (>1.2 or <0.83) and P-value <0.05 were used to identify differentially expressed proteins (DEPs). Functional annotation of all identified proteins was performed using GO (http://geneontology.org/) and KEGG pathway (http://www.genome.jp/kegg/). DEPs were further used to for GO and KEGG enrichment analysis.

Table S1. Information of sixteen DEPs for PRM analysis.

| **Protein ID** | **Name** | **Annotation** | **Peptide sequence** |
| --- | --- | --- | --- |
| A0A1Y0VUZ3 | *ldh* | L-lactate dehydrogenase | EEYGTYLSYPAVVGR |
| A0A1Y0VQW8 | *rpsB* | Small ribosomal subunit protein uS2 | DVAANDGVVLFVGTK |
| A0A0R2HAT3 | *rplA* | Large ribosomal subunit protein uL1 | VSFDVDQLLGNLQTINDTIVR |
| A0A1Y0VQZ9 | *rpmE2* | Large ribosomal subunit protein bL31B | FLSGSTVGSAETIK |
| A0A7T4MX93 | *rplI* | Large ribosomal subunit protein bL9 | AATSAAVSELK |
| A0A0R2HFB4 | *rplV* | Large ribosomal subunit protein uL22 | SVAEAFAILK |
| A0A7T4T5P2 | *polA* | 50S ribosomal protein L28 | LSSIDPNLQNIPVR |
| A0A0Q0TT21 | *pfkA* | DNA polymerase I | YPEFAQEESQLK |
| A0A6L5A1F8 | *eno* | Enolase | IEEEIGVSDAR |
| A0A0Q0YZ86 | *tpiA* | Triosephosphate isomerase | NPQETQEFLDAIK |
| A0A0R2H759 | *rpsQ* | Small ribosomal subunit protein uS17 | TITVQIDTYK |
| A0A0R2HGS7 | *rplO* | Large ribosomal subunit protein uL15 | GFTNINR |
| A0A0R2H6Y7 | *rplW* | Large ribosomal subunit protein uL23 | AIEEIFEVTVIK |
| A0A1Y0VUP1 | *polC* | DNA polymerase III PolC-type | FLYPTLK |
| A0A7T4MYD6 | *recJ* | Single-stranded-DNA-specific exonuclease | FELEDVR |
| A0A1Y0VWI4 | *accA* | acetyl-CoA carboxytransferase | IVPEVVDQTSTQNLK |

**Supporting Results**

**3.1 Characteristics of nettle silage after inoculation with *P. pentosaceus***

Figure S1. Phylogenetic evolution tree of *Pediococcus* isolated from nettle silage inferred from the Neighbour-joining method. The number displayed on the branches shows the percentage of closeness, tree shows a closer relatedness of the identified species (OL455604) to *Pediococcus pentosaceus.*


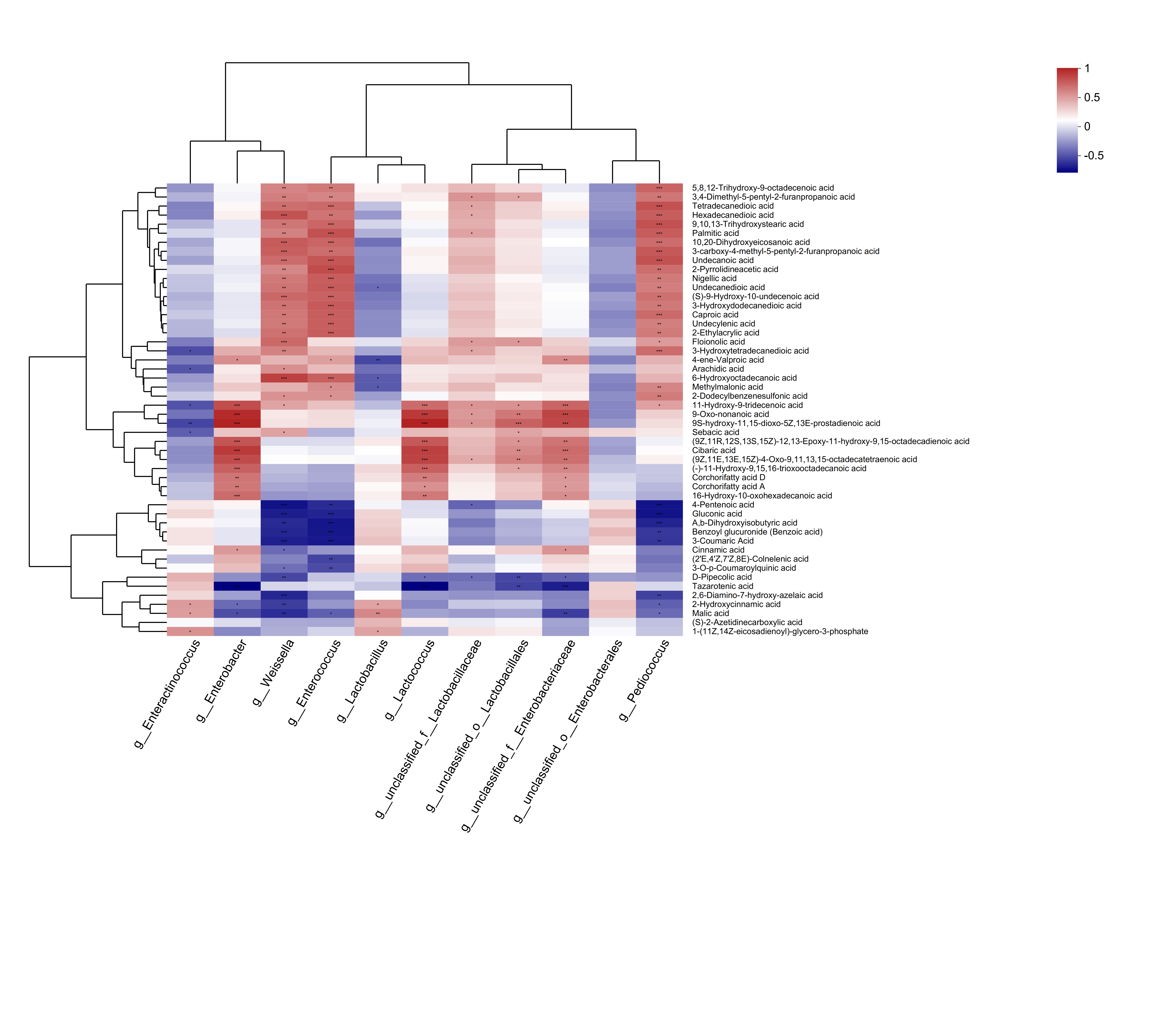


Figure S2. Correlation between bacteria and metabolites in naturally fermentation of nettle silage. “*” mean 0.01＜*p*＜0.05; “**” mean 0.001＜*p*＜0.01; “***” mean *p*＜0.001, respectively.


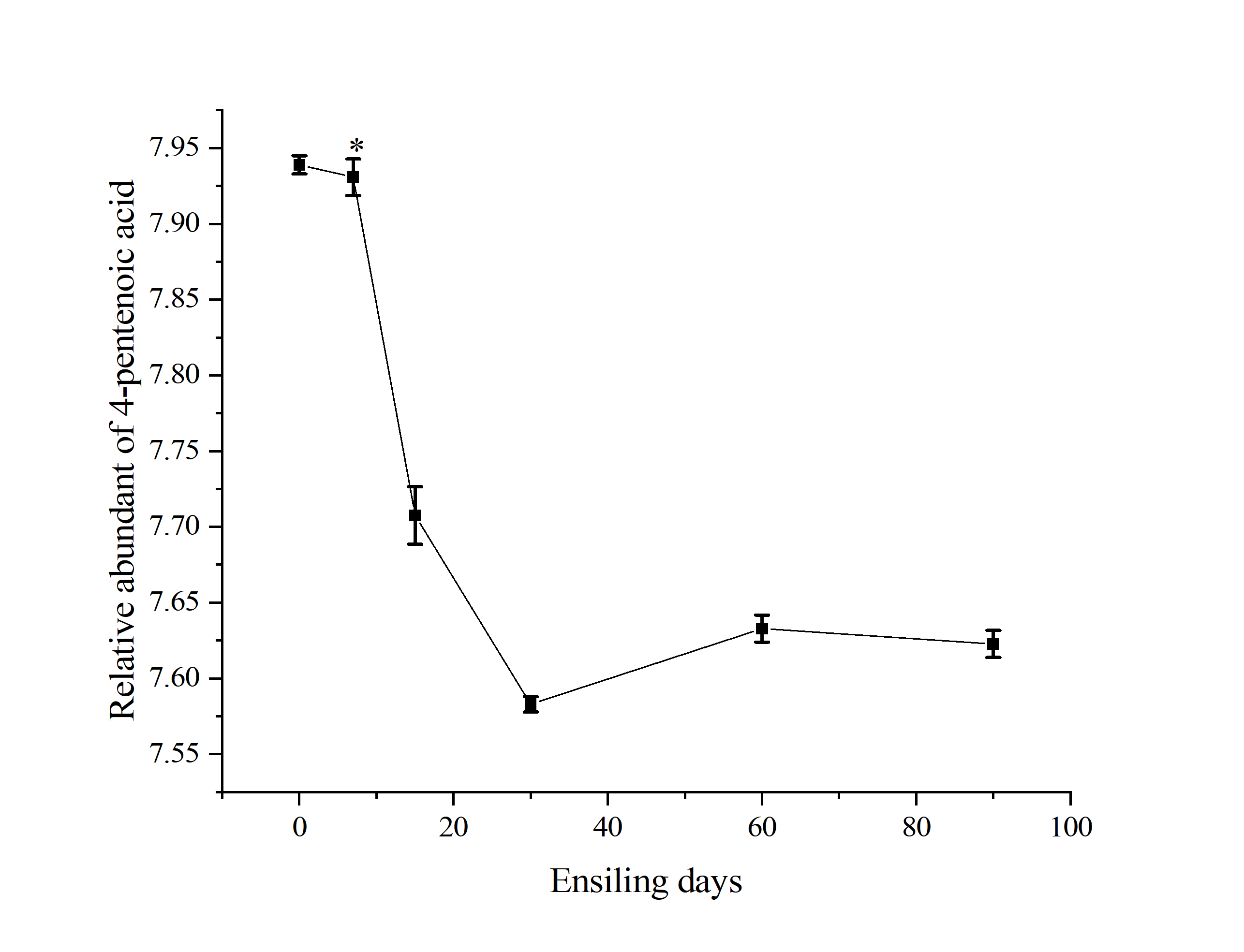


Figure S3. Relative abundant of 4-pentenoic acid during nettle ensiling. “*” means significant difference with others as *p*＜0.05

**3.3 Effect of 4-pentenoic acid on *P. pentosaceus* growth**


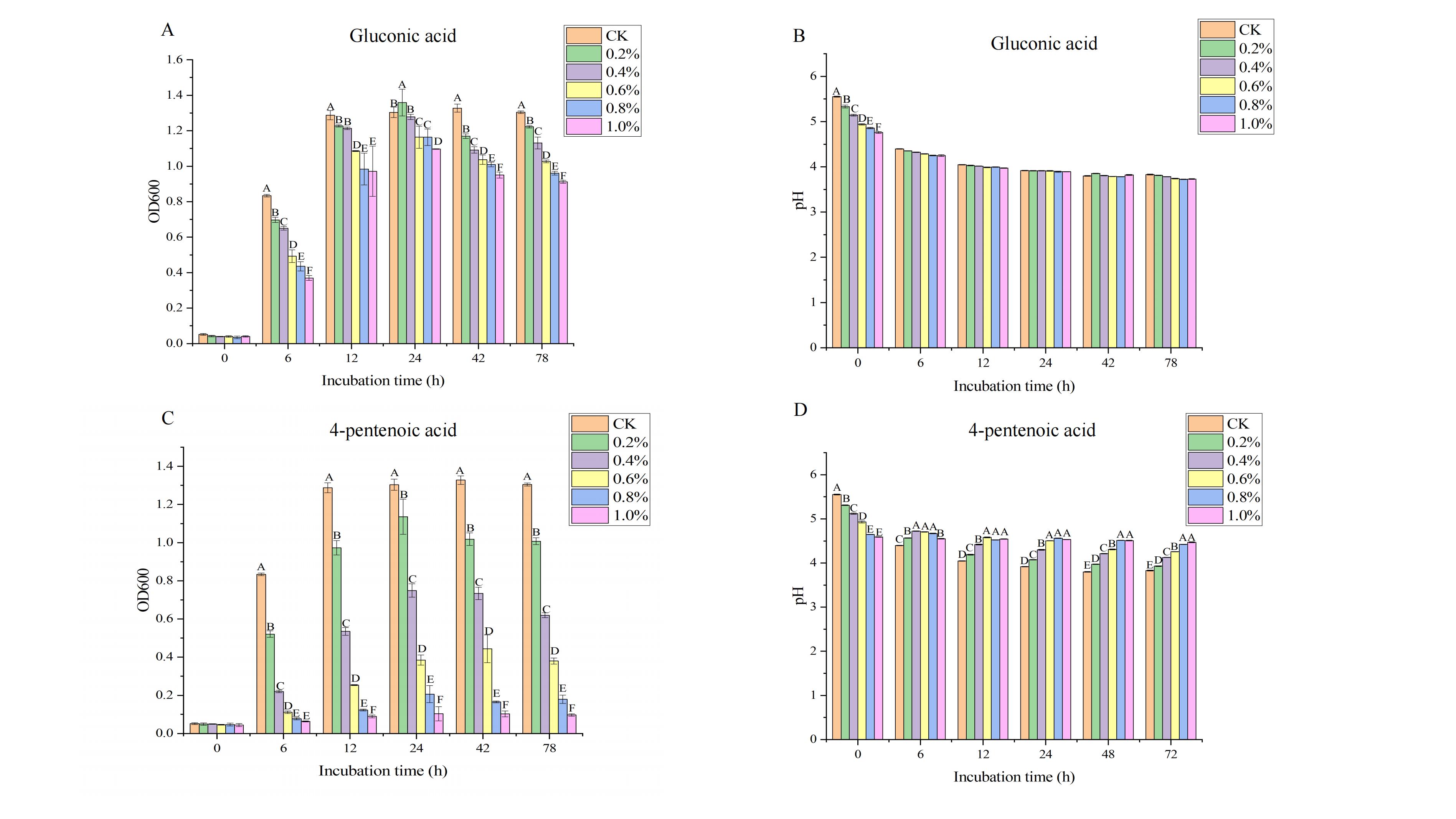


Figure S4. Effect of organic acid on *P. pentosaceus* growth with gluconic acid (A) OD600nm (B) pH and 4-pentenoic acid (C) OD600nm (D) pH, respectively. CK: control group, 0.2%: addition with organic acid at 0.2% (v: v) in MRS medium, same as others. Different capital letters on each bar means significant difference (*p*＜0.05).

**3.4 Proteomic profiling in *P. pentosaceus* under 4-pentenoic acid stress**

Table S2 Results of sixteen DEPs for PRM analysis

| **Protein ID** | **Name** | **Annotation** | **Log2 FC (DT/CK)** | |
| --- | --- | --- | --- | --- |
|  |  |  | PRM | Proteo-mic |
| A0A1Y0VUZ3 | *ldh* | L-lactate dehydrogenase | -1.94 | 0.89 |
| A0A1Y0VQW8 | *rpsB* | Small ribosomal subunit protein uS2 | -2.38 | -0.97 |
| A0A0R2HAT3 | *rplA* | Large ribosomal subunit protein uL1 | -5.03 | -1.12 |
| A0A1Y0VQZ9 | *rpmE2* | Large ribosomal subunit protein bL31B | -3.16 | -1.46 |
| A0A7T4MX93 | *rplI* | Large ribosomal subunit protein bL9 | -3.58 | -1.62 |
| A0A0R2HFB4 | *rplV* | Large ribosomal subunit protein uL22 | -3.35 | -0.82 |
| A0A7T4T5P2 | *polA* | 50S ribosomal protein L28 | -4.19 | -0.68 |
| A0A0Q0TT21 | *pfkA* | DNA polymerase I | -2.11 | -0.67 |
| A0A6L5A1F8 | *eno* | Enolase | 0.94 | 1.81 |
| A0A0Q0YZ86 | *tpiA* | Triosephosphate isomerase | -1.20 | 1.09 |
| A0A0R2H759 | *rpsQ* | Small ribosomal subunit protein uS17 | -3.46 | -0.95 |
| A0A0R2HGS7 | *rplO* | Large ribosomal subunit protein uL15 | -3.95 | -2.04 |
| A0A0R2H6Y7 | *rplW* | Large ribosomal subunit protein uL23 | -2.75 | -0.65 |
| A0A1Y0VUP1 | *polC* | DNA polymerase III PolC-type | -4.14 | -0.74 |
| A0A7T4MYD6 | *recJ* | Single-stranded-DNA-specific exonuclease | -4.62 | -0.74 |
| A0A1Y0VWI4 | *accA* | acetyl-CoA carboxytransferase | -2.71 | -0.76 |

DT: 4-pentenoic acid treated group, CK: control group.


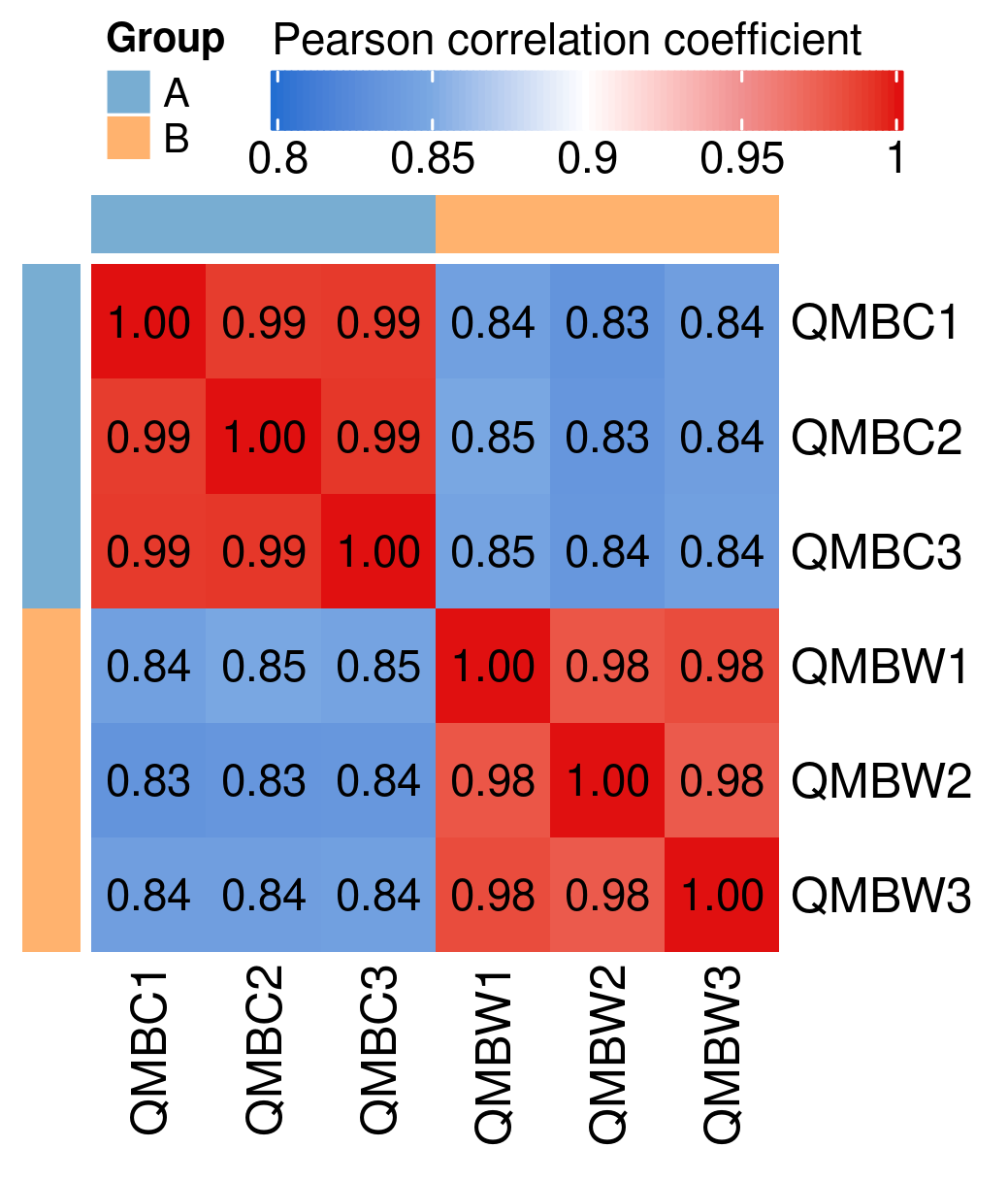


Figure S5. Pearson's correlation between the *P. pentosaceus* cells treated without (QMBC) and with 0.8 % (v/v) 4-pentenoic acid (QMBW) for 8 h.

**Reference**

Keerthana, C., and Narayanan, R. B. (2021). Identification and Characterization of Pediococcus Species from Piper betle (Betel) Leaves. *Current Microbiology* 78, 198–205. doi: 10.1007/s00284-020-02270-2

Schloss, P. D., Westcott, S. L., Ryabin, T., Hall, J. R., Hartmann, M., Hollister, E. B., et al. (2009). Introducing mothur: Open-Source, Platform-Independent, Community-Supported Software for Describing and Comparing Microbial Communities. *Applied And Environment Microbiology* 75, 7531–7541. doi: 10.1128/AEM.01541-09

Sun, Y., Sun, Q. L., Tang, Y. M., Li, Q. Y., Tian, C. J., and Sun, H. X. (2023). Integrated microbiology and metabolomic analysis reveal the improvement of rice straw silage quality by inoculation of Lactobacillus brevis. *Biotechnology for Biofuels and Bioproducts* 16, 184. doi: 10.1186/s13068-023-02431-y
